# A novel *GJA8* mutation (p.I31T) causing autosomal dominant congenital cataract in a Chinese family

**Published:** 2009-12-16

**Authors:** Kaijie Wang, Binbin Wang, Jing Wang, Shiyi Zhou, Bo Yun, Peisu Suo, Jie Cheng, Xu Ma, Siquan Zhu

**Affiliations:** 1 Beijing Tongren Eye Center, Beijing Tongren Hospital, Capital Medical University, Beijing Ophthalmology & Visual Sciences Key Lab, Beijing, China; 2 National Research Institute for Family Planning, Beijing, China; 3 Peking Union Medical College, Beijing, China; 4 World Health Organization Collaborating Center for Research in Human Reproduction, Beijing, China

## Abstract

**Purpose:**

To identify the genetic defect associated with autosomal dominant congenital nuclear cataract in a Chinese family.

**Methods:**

Family history and clinical data were recorded. The genomic DNA was extracted from peripheral blood leukocytes. All the members were genotyped with microsatellite markers at loci considered to be associated with cataracts. Two-point logarithm of odds (LOD) scores were calculated by using the Linkage software after genotyping. Mutations were detected by DNA sequence analysis of the candidate genes. Effects of amino acid changes on the structure and function of proteins were predicted by bioinformatics analysis.

**Results:**

Evidence of a linkage was obtained at markers D1S514 (LOD score [Z]=3.48, recombination fraction [θ]=0.0) and D1S1595 (Z=2.49, θ=0.0). Haplotype analysis indicated that the cataract gene was close to these two markers. Sequencing of the connexin 50 (*GJA8*) gene revealed a T>C transition at nucleotide position c.92. This nucleotide change resulted in the substitution of highly conserved isoleucine by threonine at codon 31(I31T). This mutation co-segregated with all affected individuals and was not observed in unaffected or 110 normal unrelated individuals. Bioinformatics analysis showed that a highly conserved region was located at Ile31, and the mutation was predicted to affect the function and secondary structure of the GJA8 protein.

**Conclusion:**

A novel mutation in *GJA8* was detected in a Chinese family with autosomal dominant congenital nuclear cataract, providing clear evidence of a relationship between the genotype and the corresponding cataract phenotype.

## Introduction

Congenital cataract is a clinically and genetically heterogeneous group of eye disorders that causes visual impairment and childhood blindness. Its prevalence is up to 6 in 10,000 live births, causing about 10% of childhood blindness worldwide [1-4]. Cataracts can be isolated or can occur in association with a large number of different metabolic diseases or genetic syndromes. Nearly one-third of the cases show a positive family history. Although all three Mendelian modes of inheritance have been observed for congenital cataracts, autosomal dominant seems to be the most common mode of inheritance [5].

To date, more than 30 independent loci and 18 cataract-related genes have been identified as being associated with isolated autosomal dominant congenital cataract (ADCC). These genes can be divided into five groups, including: (1) genes encoding crystallins: *CRYAA*, *CRYAB*, *CRYBA1/A3*, *CRYBA4*, *CRYBB1*, *CRYBB2*, *CRYGC*, *CRYGD,* and *CRYGS* [6-14]; (2) genes encoding membrane transport and channel proteins: *GJA3*, *GJA8*, and *MIP* (also know as *AQP0*) [15-17]; (3) genes encoding cytoskeletal proteins, such as *BFSP2* [18]; (4) genes encoding transcription factors, such as *PITX3, HSF4*, and *MAF* [19-21]; and (5) others: *CHMP4B* [22] and *EPHA2* [23]. The same mutation in different families or even within a family can result in drastically different morphologies and severity of lens opacification. On the other hand, similar or identical cataract presentation may arise from mutations of different genes. These observations suggest that additional genes or modifying factors, such as environmental regulators, could play important roles in cataract onset, progression, and maturation.

In the present study we investigated a large Chinese family with autosomal dominant congenital nuclear cataract and detected a novel missense mutation in *GJA8* that co-segregated with the disease in the family.

## Methods

### Clinical evaluation and DNA specimens

This study adhered to the tenets of the Declaration of Helsinki and was approved by the ethics committees for medical research at Capital Medical University. A five-generation family with non-syndromic congenital cataracts was recruited at the Beijing Tongren Eye Center, Beijing Tongren Hospital (Capital Medical University, Beijing, China). The study consisted of 30 members, including 12 affected individuals, 18 unaffected individuals, originating from the province of Hebei, China. Clinical and ophthalmological examinations were performed. There was no history of other ocular or systemic abnormalities in the family. The mean age of the 13 women and 17 men was 41 years (range: 3 to 78 years). Informed consents were obtained from all participants. Affected status was determined by a history of cataract extraction or ophthalmologic examination, including visual acuity, slit lamp, and fundus examination. The phenotypes were documented by slit lamp photography. A total of 110 unrelated control subjects with no family history of congenital cataracts were also recruited. They were given complete ophthalmologic examinations as the study subjects of the cataract family and did not have eye diseases except mild myopia and senile cataracts. Peripheral venous blood was collected for genomic DNA extraction using a QIAamp DNA kit (Qiagen, Valencia, CA) according to the manufacturer’s instructions. A 200 µl aliquot of blood sample was incubated with QIAGEN protease and buffer AL at 56 °C for10 min. The lysate was applied to a QIAamp spin column, and washed twice with buffer AW and finally eluted with 200 µl of Buffer AE. The DNA was stored at -20 °C until use.

### DNA genotyping

PCRs were performed with microsatellite markers close to candidate loci associated with autosomal congenital cataracts. PCR products from each DNA sample were separated from a 6% polyacrylamide gel and examined. Pedigree and haplotype data were analyzed with Cyrillic software (ver. 2.1; Cherwell, Scientific Ltd, United Kingdom ). Exclusion analysis was performed by allele sharing in affected individuals.

### Linkage analysis

A two-point linkage was calculated with the Linkage package (ver. 5.1; provided in the public domain by Rockefeller University, New York, NY). The cataracts in this family were analyzed as an autosomal dominant trait with full penetrance and a gene frequency of 0.0001. The allele frequencies for each marker were assumed to be equal in both genders. The marker order and distances between the markers were taken from the National Center for Biotechnology Information (NCBI) database.

### Mutation analysis

All coding exons and splice sites of *GJA8* were amplified by PCR using primer pairs shown in Table 1. The PCR products were sequenced on an ABI3730 Automated Sequencer (PE Biosystems, Foster City, CA). The data were compared with sequences from the NCBI GenBank (*GJA8*: NM_005267.3).

**Table 1:** Primer sequences for *GJA8*.

**Amplicon**	**Forward primer (5′→3′)**	**Reverse primer (5′→3′)**
1	CCGCGTTAGCAAAAACAGAT	CCTCCATGCGGACGTAGT
2	GCAGATCATCTTCGTCTCCA	GGCCACAGACAACATGAACA
3	CCACGGAGAAAACCATCTTC	GAGCGTAGGAAGGCAGTGTC
4	TCGAGGAGAAGATCAGCACA	GGCTGCTGGCTTTGCTTAG

Forward and reverse primer sequences were provided for each amplicon of *GJA8*.

### Bioinformatics analysis

The possible functional impact of an amino acid change was predicted by the PolyPhen (Polymorphism Phenotyping) program. The prediction is based on the position-specific independent counts (PSIC) score derived from multiple sequence alignments of observations. PolyPhen scores of >2.0 indicate the polymorphism is probably damaging to protein function; scores of 1.5–2.0 are possibly damaging; and scores of <1.5 are likely benign. The secondary structure of mutant and wild-type amino acid sequences were analyzed by Antheprot 2000 ver. 6.0 software (IBCP, Lyon,France).

## Results

### Clinical findings

**Figure 1:**
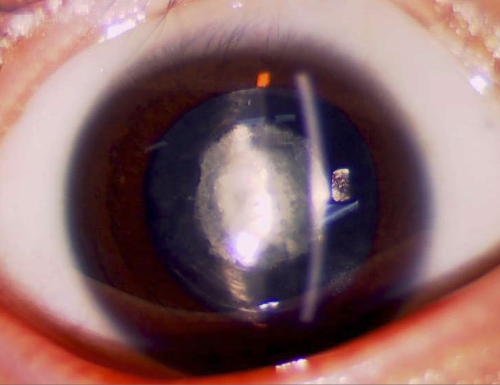
Slit lamp photographs of the proband. The photograph of the proband (V:3) shows that the opacity is a nuclear cataract. Both the fetal nucleus and the embryonic nucleus display white opacities.

We identified a five-generation Chinese family with clear diagnosis of ADCC. The affected individuals presented with bilateral congenital nuclear cataracts that consisted of a central nuclear opacity affecting the embryonic and fetal nucleus of the lens (Figure 1). According to the history and medical records, all affected individuals were diagnosed before the age of 2. The lens opacity caused obvious vision loss, visual acuity ranging from 0.06 to 0.2. Nine of the 12 patients received cataract surgery before the age of 20. There was no family history of other ocular or systemic abnormalities.

### Linkage and haplotype analysis

**Figure 2:**
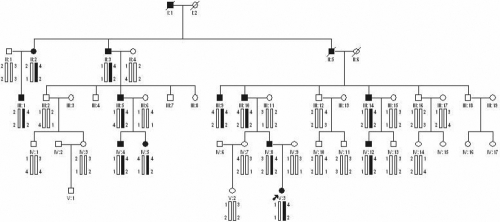
Cataract pedigree and haplotype analysis. Pedigree and haplotype analysis of the cataract family shows the segregation of two microsatellite markers on chromosome 1p12-q22. Squares and circles indicate males and females, respectively. Blackened symbols and bars denote affected status.

Allele-sharing analysis excluded the linkage of the disease in the family with all known loci of cataract except for *GJA8*. Haplotype analysis showed that the affected individuals in the family shared a common haplotype with markers D1S514 and D1S1595 at 1p12-q22 (Figure2). Significant evidence of linkage was observed with microsatellite markers D1S514 (logarithm of odds ]LOD] score [Z]=3.48, recombination fraction [θ]=0.0) and D1S1595 (Z=2.49, θ=0.0) (Table 2). This suggested that *GJA8* in the region might be responsible for the disease.

**Table 2:** two-point LOD scores for linkage between the cataract locus and 1P12-Q22 markers.

Marker	LOD scores at recombination for value of θ					
	0.0	0.1	0.2	0.3	0.4	0.5
D1S514	3.48	2.82	2.10	1.32	0.53	0.00
D1S1595	2.49	1.96	1.40	0.83	0.31	0.00

Two-point LOD score for linkage in chromosome 1 markers. The maximum two-point LOD score (3.48) was achieved at D1S514 at θ=0.

### Mutation analysis

**Figure 3:**
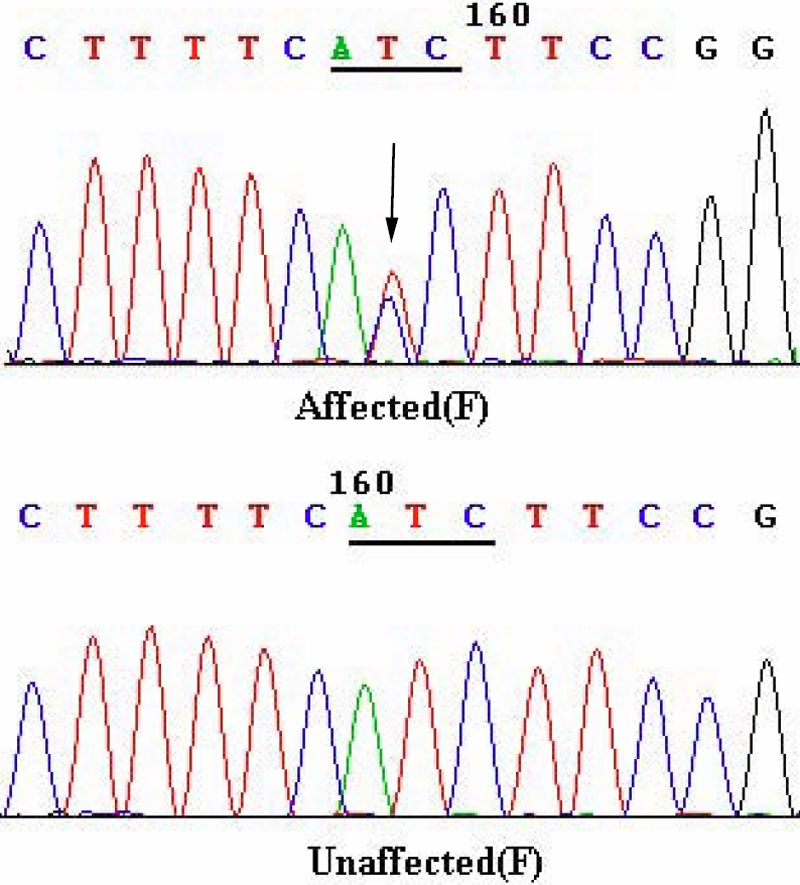
DNA sequence chromatograms. DNA sequence chromatograms of the unaffected members and affected members in an autosomal dominant nuclear cataract family. A single transition is observed at position 92 (T>C) as a T/C double peak (indicated by an arrow).

Direct cycle sequencing of the amplified fragments of *GJA8* in 12 affected individuals identified a single base alteration c.92 T>C in the exon of *GJA8* (Figure 3), which resulted in a substitution of isoleucine to threonine at codon 31 (p.I31T). The alteration was not seen in any of the unaffected family members tested nor in the 110 unrelated control subjects from the same Northern Chinese population (data not shown) but was confirmed in all affected individuals.

### Bioinformatics analysis

**Figure 4:**
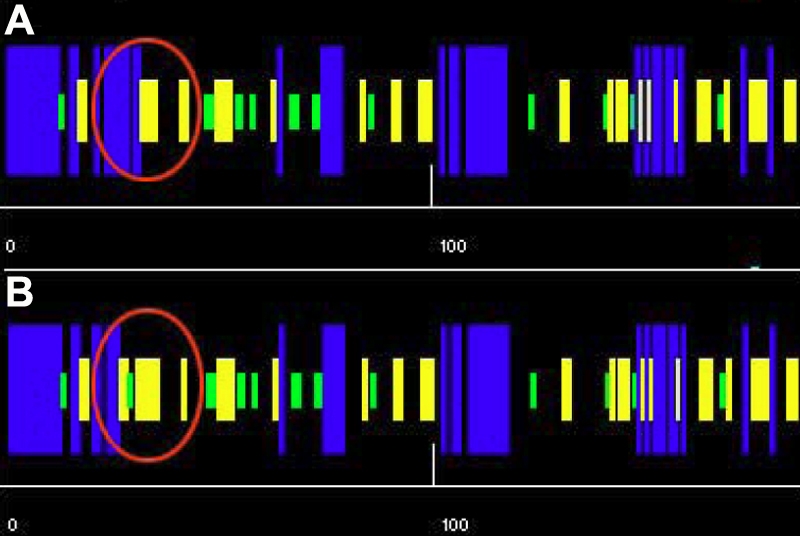
The predicted secondary structures of the mutant and the wild-type amino acid sequences. The predicted secondary structures of the wild-type amino acid sequence (**A**) and the mutant amino acid sequence (**B**) is shown. The target sequences are labeled by a red circle, which indicate that there is a helix in the wild type replaced by a turn in the mutant type. Blue, helix; yellow, sheet; green, turn; black, coin.

**Figure 5:**
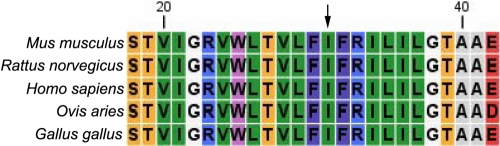
Multiple-sequence alignment in *GJA8* from different species. A multiple alignment of partial amino acid sequences of *GJA8* from different species is shown. The alignment data indicate that isoleucine at position 31 (indicated by an arrow) is highly conserved in different species in *GJA8*.

The Polyphen score from Polyphen analysis was 2.334, which means that this I31T GJA8 was predicted with high confidence to be “possibly damaging.” The secondary structure prediction showed that the mutation I31T led to the replacement of an original helix by a turn, a significant difference in coding position 31 of the secondary structure of GJA8 (Figure 4). The place where the mutation occurred was located within a phylogenetically conserved region by multiple-sequence alignment (Figure 5).

## Discussion

The nuclear cataract characterized in this study was associated with a mutation in the *GJA8* gene encoding connexin 50, a gap junction protein expressed in lens fiber cells. The lens is an avascular structure, and lens fibers lose all intracellular organelles during development. The lens has developed an extensive cell–cell communication system using connexins to maintain its transparency [24]. Gap junction intercellular communication is an essential part of this system, which facilitates the exchange of ions, metabolites, signaling molecules, and other molecules with a molecular weight up to 1 kDa [25].

Connexins are integral membrane proteins containing four transmembrane domains, two extracellular loops, and an intracellular loop with both the amino and carboxyl termini located in the cytoplasm. The coding region of connexin 50 is completely contained within one exon, which encodes a polypeptide containing 432 amino acids with a molecular weight of 48,171 Da. The observed p.I31T substitution is located within the first transmembrane domain (M1) of connexin 50 and replaces the highly conserved nonpolar isoleucine by polar threonine at position 31 in association with the congenital cataract in the present study. The transmembrane domains of the connexins are proposed to participate in the oligomerization into connexon hemichannels and are also essential for the correct transport of the protein into the plasma membrane. The pore lining residues have been identified as lying in the first transmembrane domain and are essential for the formation of the pore, and therefore channel, permeability [26].

Isoleucine-31 is well conserved in the first transmembrane domain of connexin 50 in different species by multiple-sequence alignment. The I31T mutation is predicted by Polyphen analysis to be possibly damaging, which highlights the functional importance of this region of the protein. The secondary structure of the mutant protein is predicted, and the helix is replaced by a turn, which may be the reason for the dysfunction of the mutant protein. The mutation may influence the correct transport of proteins into the plasma membrane. This would contribute to abnormal intercellular communication in the lens and would result in nuclear opacification of the lens.

Mutations in the *GJA8* gene have been demonstrated to be one of the most frequent reasons for isolated congenital cataracts. Different mutations have been detected in *GJA8*, and significant interfamilial phenotypic variability has been observed (Table 3). To date, at least 18 congenital cataract families have been linked with *GJA8*, and most mutations detected affect the first half of the protein. The phenotypes in most of the cases have been described as nuclear pulverulent cataract. In the present family, the phenotype observed also showed a marked nuclear cataract but without microcornea, comparable with the mutations in M1 of *GJA8*, which are all linked with cataract and microcornea in another two Indian families (Table 3). Functional studies on the mutation of *GJA8* polypeptides have shown diverse mutational mechanisms, such as a dominant negative effect and loss-of-function of the mutant protein [27,30,35]. Functional implications of these mutations may account for the phenotypic differences.

**Table 3:** Summary of identified mutations in *GJA8*.

**Mutation**	**Amino acid change**	**Location**	**Cataract type**	**Origin of family**	**Reference**
68G>C	R23T	NH_2_-terminus	Progressive dense nuclear	Iranian	[27]
92T>C	I31T	First transmembrane domain (M1)	Nuclear cataract	Chinese	Present study
131T>A	V44E	First transmembrane domain (M1)	Cataract and microcornea	Indian	[28]
134G>C	W45S	First transmembrane domain (M1)	Jellyfish-like cataract and microcornea	Indian	[29]
139G>A	D47N	First extracellular loop (E1)	Nuclear pulverulent cataract	English	[30]
139G>T	D47Y	First extracellular loop (E1)	Nuclear cataract	Chinese	[31]
142G>A	E48K	First extracellular loop (E1)	Zonular nuclear pulverulent	Pakistani	[32]
191T>G	V64G	First extracellular loop (E1)	Nuclear cataract	Chinese	[33]
235G>C	V79L	Second transmembrane domain (M2)	Full moon like with Y-sutural opacities	Indian	[34]
262C>T	P88S	Second transmembrane domain (M2)	Zonular pulverulent	English	[16]
262C>A	P88Q	Second transmembrane domain (M2)	Lamellar pulverulent	English	[35]
262C>A	P88Q	Second transmembrane domain (M2)	“Balloon-like”cataract with Y-sutural opacities	Indian	[36]
565C>T	P189L	Second transmembrane domain (M2)	Nuclear cataract and microcornea	Danish	[37]
593G>A	R198Q	Second transmembrane domain (M2)	Posterior subcapsular cataract and microcornea	Indian	[28]
670insA	fs	Second transmembrane domain (M2)	Total cataract and nystagmus	Indian	[38]
741T>G	I247M	COOH-terminus	Zonular pulverulent cataract	Russian	[39]
ins776G	fs	COOH-terminus	Triangular nuclear cataract	German	[40]
827C>T	S276F	COOH-terminus	Pulverulent nuclear cataract	Chinese	[41]

Shown are *GJA8* gene mutations that have been identified in this and other studies.

Cataractogenesis has been observed in both connexin 46 and connexin 50 knockout mice. In contrast to the connexin 46 knockout, the target ablation of connexin 50 mice resulted in nuclear cataracts in combination with smaller lenses [42,43]. Targeted replacement of connexin 50 with connexin 46 prevented cataract but did not restore normal ocular growth, which suggests that connexin 46 cannot substitute for connexin 50 in lens growth [44]. All these results clearly reveal that the *GJA8* gene plays an important role in lens fiber cell development and maintenance of transparency.

In summary, we describe a novel heterozygous I31T mutation in *GJA8* in an autosomal dominant congenital cataract family of Chinese origin. Our results further confirm that *GJA8* is important in the maintenance of optical clarity. Further study is needed to elucidate the pathophysiological consequences of this newly identified mutation in relation to the pathogenesis of cataract.
